# Supplementary material for: Immune pathways and TP53 missense mutations are associated with longer survival in canine osteosarcoma
Source: Commun Biol. 2021 Oct 11;4:1178. doi: 10.1038/s42003-021-02683-0 (PMC8505454; doi:10.1038/s42003-021-02683-0)
Supplement: Supplementary file 2 — Supplementary Information [file 42003_2021_2683_MOESM2_ESM.pdf]

Immune pathways and *TP53* missense mutations are associated with longer survival in canine osteosarcoma

Sunetra Das, Rupa Idate, Daniel P. Regan, Jared S. Fowles, Susan E. Lana, Douglas H. Thamm, Daniel L. Gustafson, Dawn L. Duval

Supplementary Information

Supplementary Tables 1-4

Supplementary Figures 1-16

Supplementary References

Supplementary Note 1

Supplementary Table 1. General description on the normal bone samples used for this study for microarray analysis.

Sample ID	Sex	Breed	Date of birth	Tumor location	Normal bone location	Neutered status
N1	Female	Labrador Retriever	6/15/96	Proximal right tibia	Proximal right femur	Yes
N2	Male	Mix	9/29/99	Distal femur	Distal tibia	Yes
N3	Female	Labrador Retriever	2/14/00	Distal left tibia	Proximal left femur	Yes
N4	Female	Canadian Husky, Red	11/1/01	Left distal femur	Distal tibia	Yes
N5	Female	Labrador Retriever	1/1/00	Proximal right humerus	Distal right radius/ulna	Yes
N8	Male	Mix	9/1/03	Proximal left humerus	Distal left radius/ulna	Yes
N9	Female	Coonhound	5/24/01	Distal left radius	Proximal left humerus	Yes
N10	Male	Labrador Retriever	3/19/03	Proximal right humerus	Distal right radius/ulna	Yes

Supplementary Table 2. The marker genes assigned to immune cell type used in this study. The gene list was derived from Rooney et al. 2015.

Immune Cell Type/ Response	Number of genes in Rooney et al. 2015	Number of genes with expression data in this canine study	Missing Orthologs	Genes analyzed in this canine study
B cells	10	9	FCRL3	CD79B, BTLA, BANK1, CD79A, BLK, RALGPS2, FCRL1, HVCN1, BACH2
CD4+ Regulatory T cells	7	5	C15orf53, IL32	FOXP3, IL5, CTLA4, GPR15, IL4
CD8+ T cells	1	1	na	CD8A
Co-inhibition, APC	5	4	C10orf54	PDCD1LG2, CD274, LGALS9, PVRL3
Co-inhibition, T cell	10	9	C10orf54	LAG3, CTLA4, CD274, CD160, BTLA, LAIR1, HAVCR2, CD244, TIGIT
Co-stimulation, APC	11	11	na	ICOSLG, CD70, TNFSF14, CD40, TNFSF9, TNFSF4, TNFSF15, TNFSF18, TNFSF8, SLAMF1, CD58
Co-stimulation, T cell	13	13	na	ICOS, CD28, CD27, TNFSF14, CD40LG, TNFRSF9, TNFRSF4, TNFRSF25, TNFRSF18, TNFRSF8, SLAMF1, CD2, CD226
Cytolytic Activity	2	2	na	GZMA, PRF1
Macrophages	10	8	C11orf45, CD68	FUCA1, MMP9, LGMN, HS3ST2, TM4SF19, CLEC5A, GPNMB, CYBB
MHC Class I	3	2	HLA-A	B2M, TAP1
Neutrophils	10	8	MEGF9, VNN3	KDM6B, HSD17B11, EVI2B, MNDA, SELL, NLRP12, PADI4, TRANK1
NK cells	2	1	KLRC1	KLRF1
pDCs	10	6	LILRA4, CLEC4C, PTCRA, GZMB	PLD4, PHEX, IL3RA, IRF8, IRF7, CXCR3
Type I IFN Response	10	9	IFIT3	MX1, TNFSF10, RSAD2, IFIT1, IFIT2, IRF7, DDX4, MX2, ISG20
Type II IFN Response	3	3	na	GPR146, SELP, AHR

Supplementary Table 3. Immune genes significantly correlated with cytolytic activity.

Cell type (Rooney et al 2015)	Corresponding marker gene	Correlation Coefficient	p value	q value
Co-stimulation, T cell	CD2	0.8156	<0.0001	<0.0001
CD8+ T cells	CD8A	0.7906	<0.0001	0.0001
Co-inhibition, T cell	CD160	0.7862	<0.0001	0.0001
Macrophages	CLEC5A	0.7535	<0.0001	0.0005
MHC Class I	TAP1	0.7313	<0.0001	0.0010
Type I IFN Response	ISG20	0.6746	0.0002	0.0042
pDCs	CXCR3	0.6708	0.0002	0.0046
Macrophages	CYBB	0.6684	0.0002	0.0048
pDCs	IRF8	0.6349	0.0005	0.0095
Type II IFN Response	AHR	0.634	0.0005	0.0096
Co-inhibition, APC	PDCD1LG2	0.6203	0.0007	0.0122
Neutrophils	EVI2B	0.6208	0.0007	0.0122
pDCs	IRF7	0.6182	0.0008	0.0127
Type I IFN Response	IRF71	0.6182	0.0008	0.0127
Co-stimulation, APC	TNFSF8	0.5943	0.0014	0.0192
B cells	CD79B	0.5892	0.0015	0.0207
Type I IFN Response	TNFSF10	0.5705	0.0023	0.0277
MHC Class I	B2M	0.5654	0.0026	0.0298
Co-inhibition, T cell	LAIR1	0.5566	0.0031	0.0335
Type I IFN Response	MX2	0.5362	0.0047	0.0431
NK cells	KLRF1	0.5359	0.0048	0.0433

Supplementary Table 4. Canine OSA cell line source and metadata.

Cell Line Name	Source (Reference index)	Breed	Sex	Xenograft
Abrams	CSU/UWM(1)	Not collected	Not collected	Yes
D-17	ATCC(2)	Poodle	Female	Yes
Gracie	UWM(3) (E.G. MacEwen)	Not collected	Not collected	Yes
HMPOS	Tokyo(4)	Mixed Breed	Male	NA
McKinley	CSU(3)	Not collected	Not collected	Yes
Moresco	UWM(3) (E.G. MacEwen)	Not collected	Not collected	NA
OS2.4	WSU(5) (D. Kochevar)	Not collected	Not collected	NA
OSA8	UM(6) (J. Modiano)	Not collected	Not collected	NA

CSU: Colorado State University.
UWM: University of Wisconsin-Madison.
OSU: The Ohio State University.
Tokyo: University of Tokyo.
WSU: Washington State University.
UM: University of Minnesota.

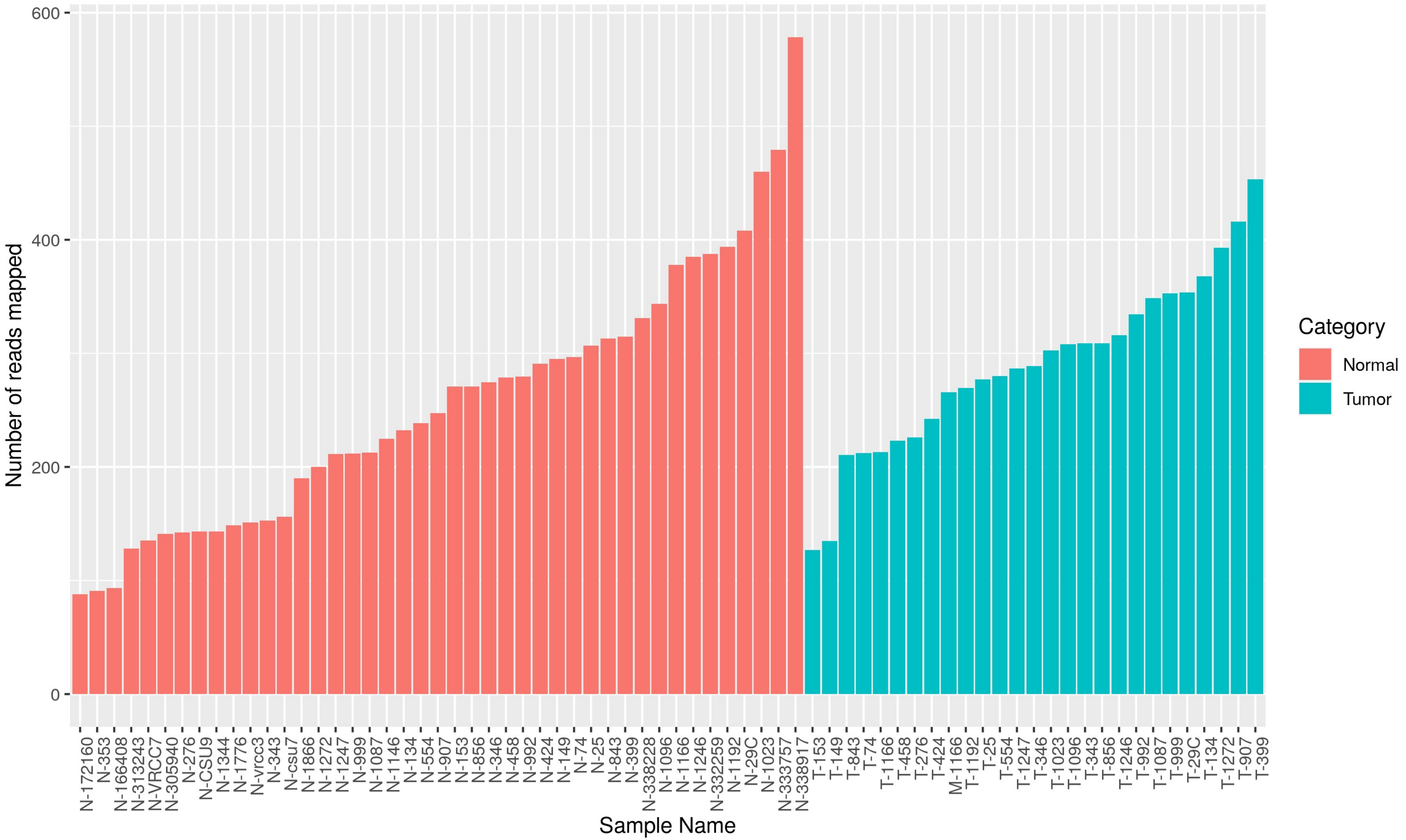

Supplementary Figure 1. Depth of sequencing across normal (N-) and tumor samples (T-). Overall, we analyzed whole exome sequence data from 43 normal and 26 osteosarcoma samples. Source data for this plot is included in the Supplementary Data 11.

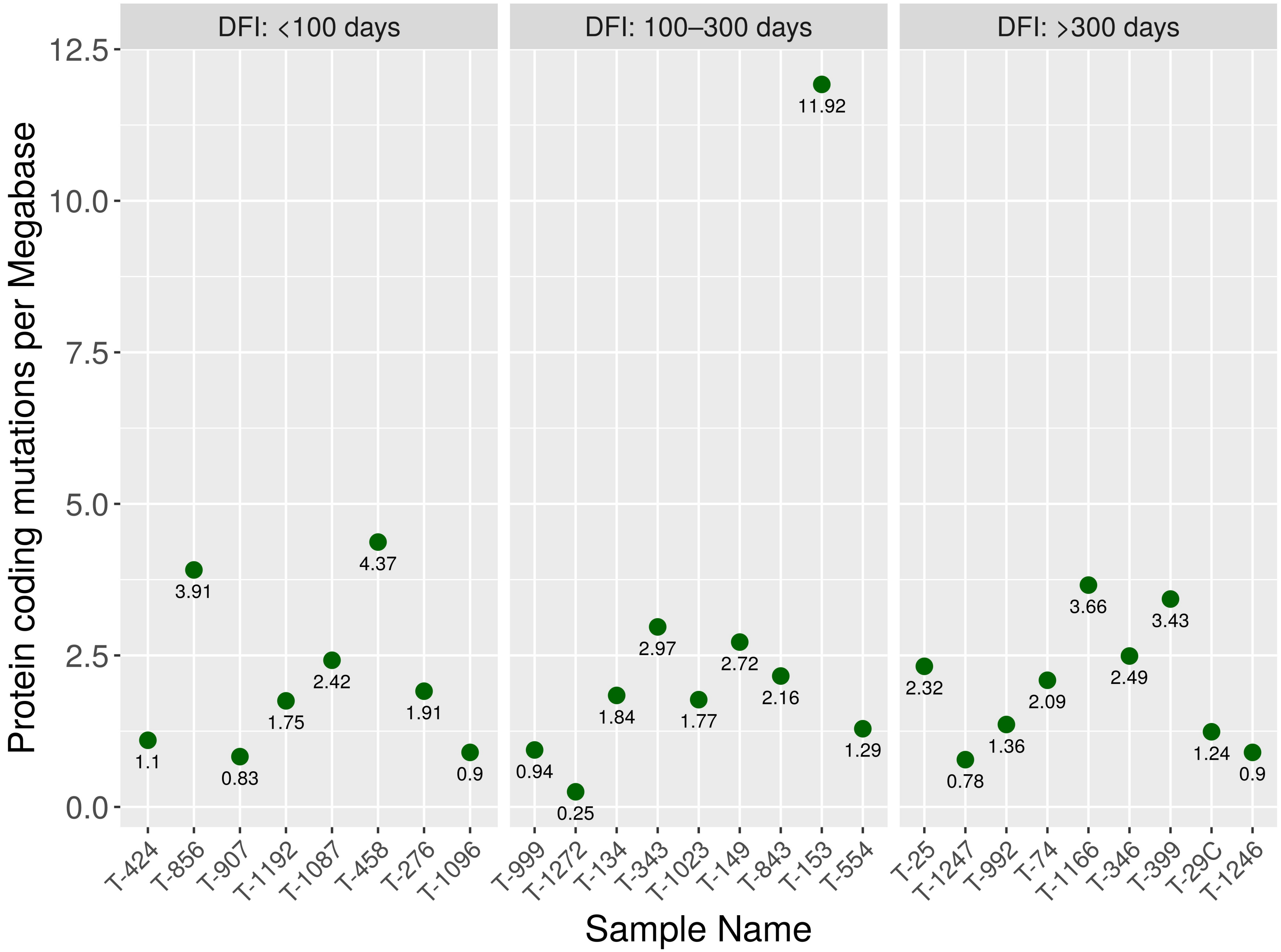

Supplementary Figure 2. Mutation burden across 26 canine osteosarcoma samples. Mutation burden is calculated as ratio of short variants over exome capture length in megabases. The samples are sorted in ascending order of disease-free interval (DFI). Source data for this plot is included in the Supplementary Data 11.

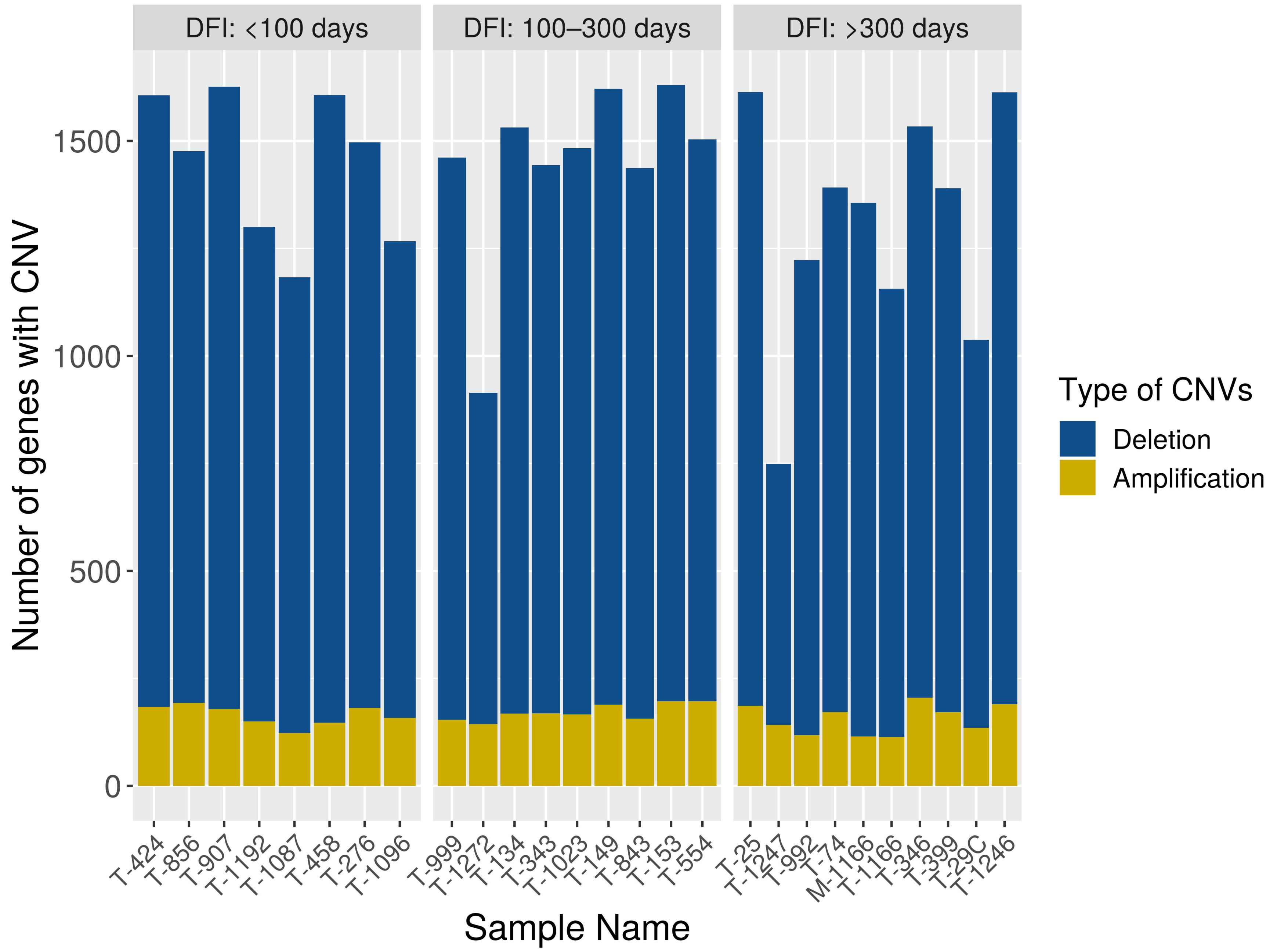

Supplementary Figure 3. Total number of copy number variants across 26 samples. Using GISTIC2.0, the significantly recurrent genes (q <0.2) within the copy number variation regions were identified. Source data for this plot is included in the Supplementary Data 11.

a

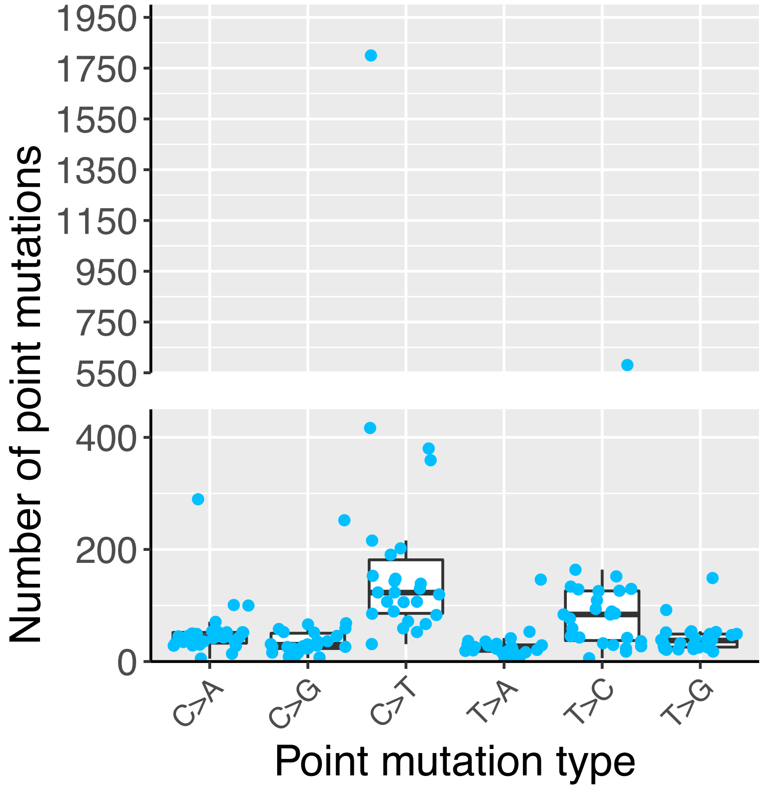

b

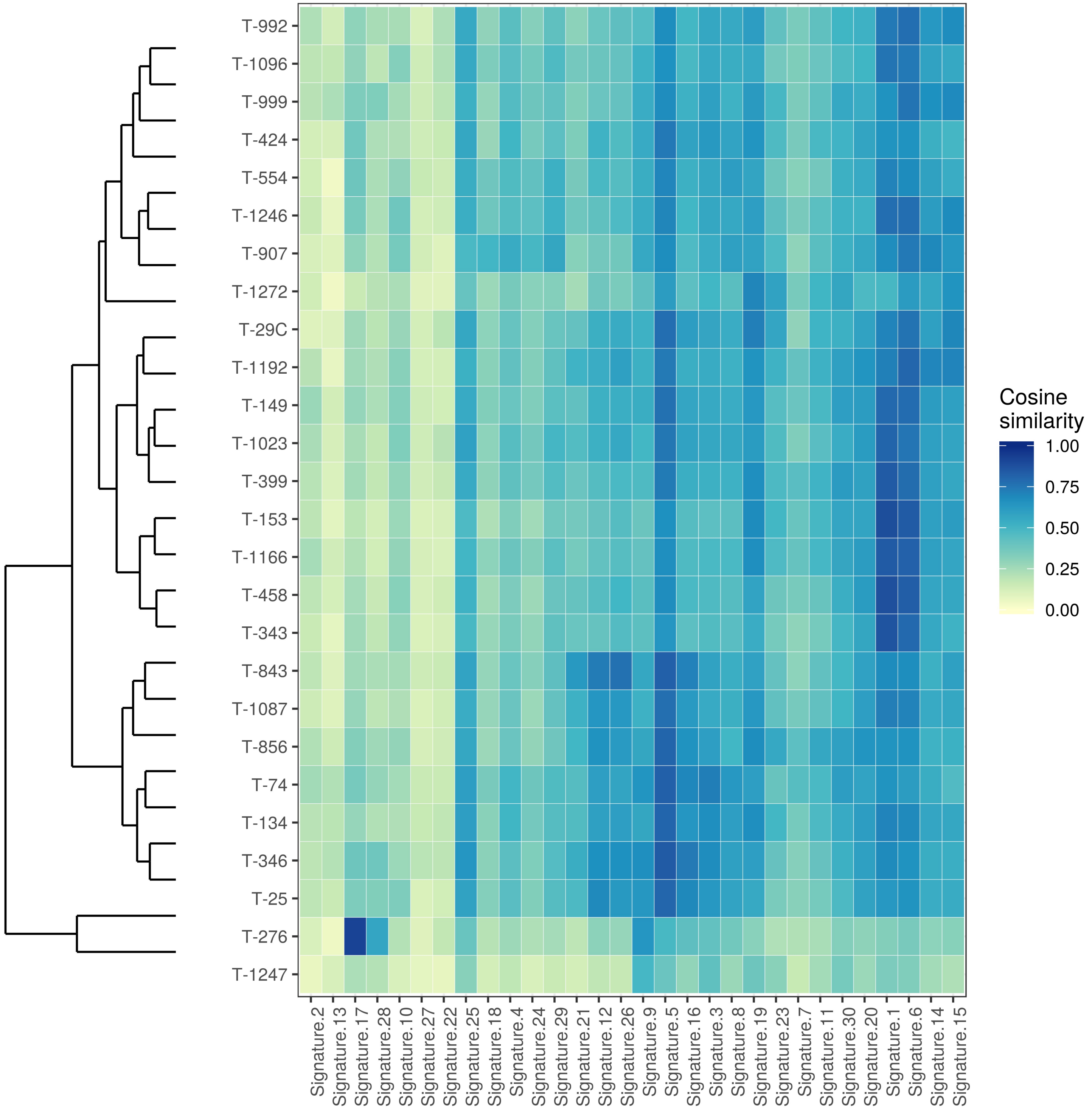

Supplementary Figure 4. Mutational signature analysis. S4a. Number of point mutations distributed across six types of single nucleotide substitution. Each point mutation types has values for 26 samples. The horizontal bar in each boxplot is its corresponding median value. S4b. Heatmap of cosine similarities between 30 known COSMIC signatures (hierarchical clustering) and 26 osteosarcoma sample mutational signatures. Source data for these plots are included in the Supplementary Data 11.

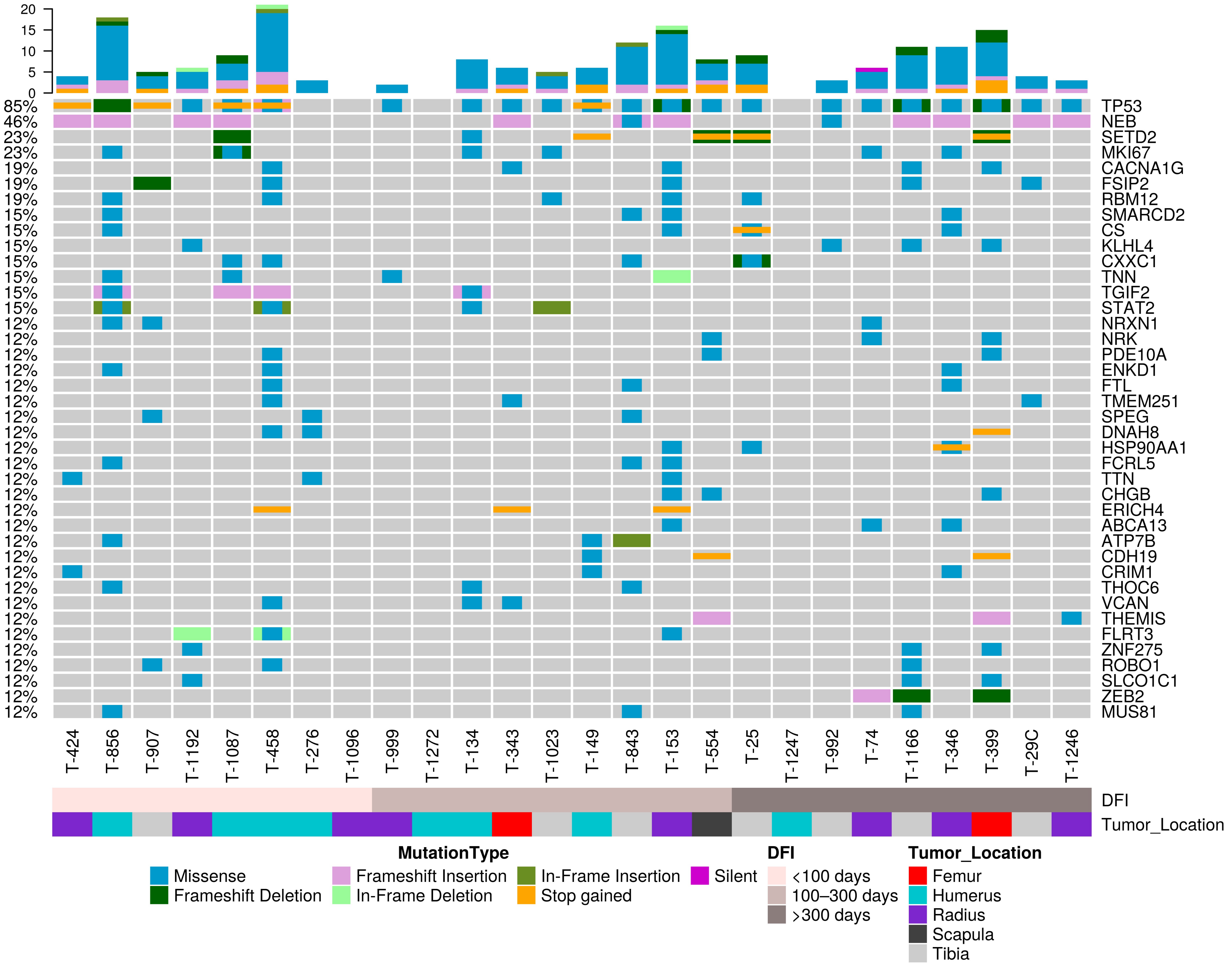

Supplementary Figure 5. Oncoplot of top 40 protein coding genes, including cancer census genes. The top histogram represents number of somatic variants in each sample. The bottom two bars represent categorization of samples based on DFI and tumor location.

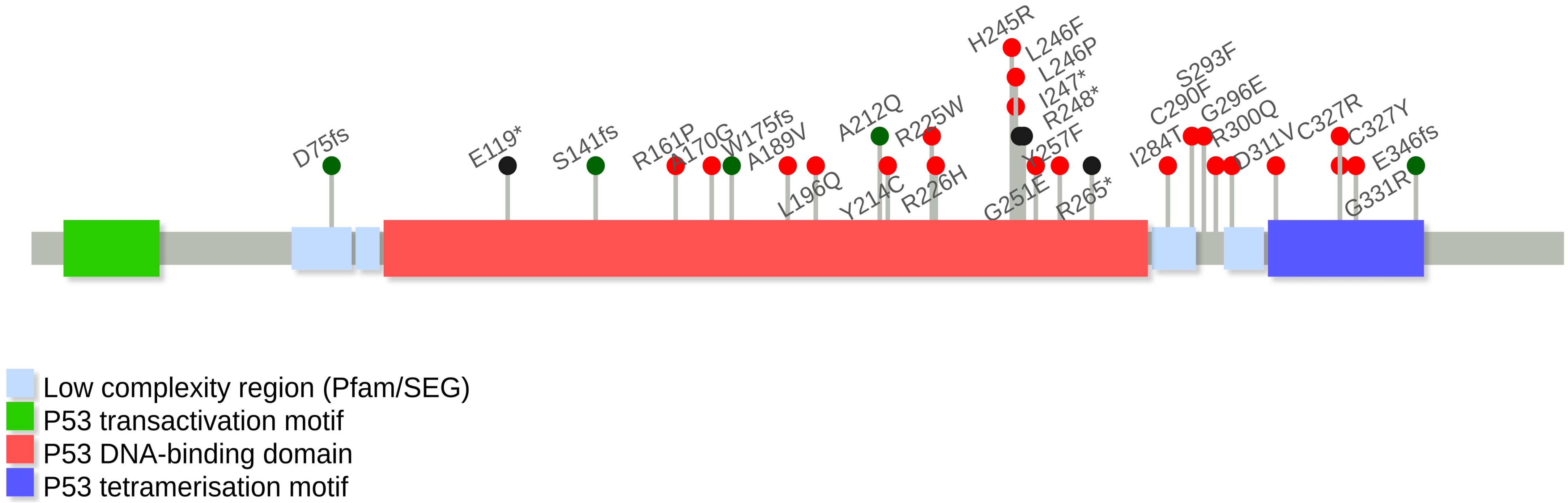

Supplementary Figure 6. TP53 mutations (Uniprot ID: A0A5F4DFH1). Lollipop plot of variant location within the TP53 protein domains.

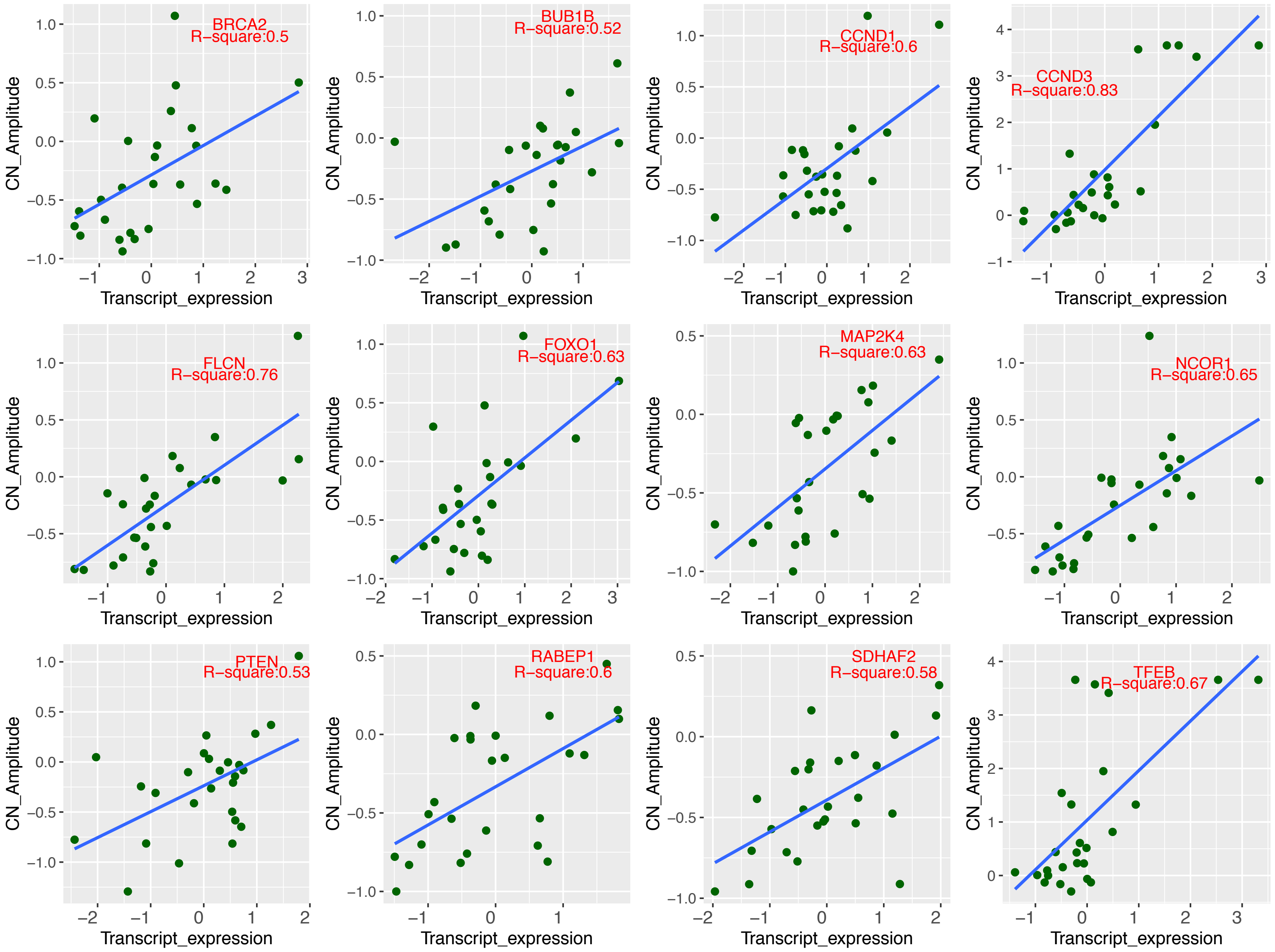

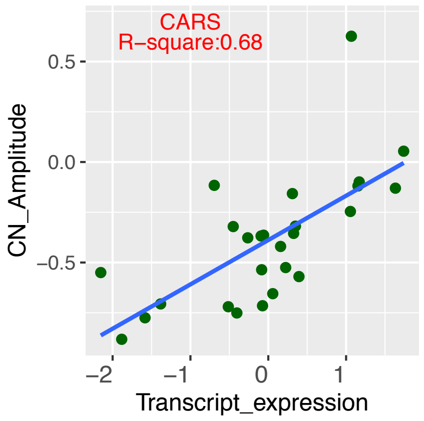

Supplementary Figure 7. Correlation of CNV amplitude and gene expression. The cancer genes with significant Pearson correlation ($p<0.05$) between transcript expression (X axis: Z-scored $\log_2$ expression) and copy number amplitude (Y axis: amplitude of change in normalized copy-number), as derived from CNV analysis, are plotted here. Source data for this plot is included in the Supplementary Data 11.

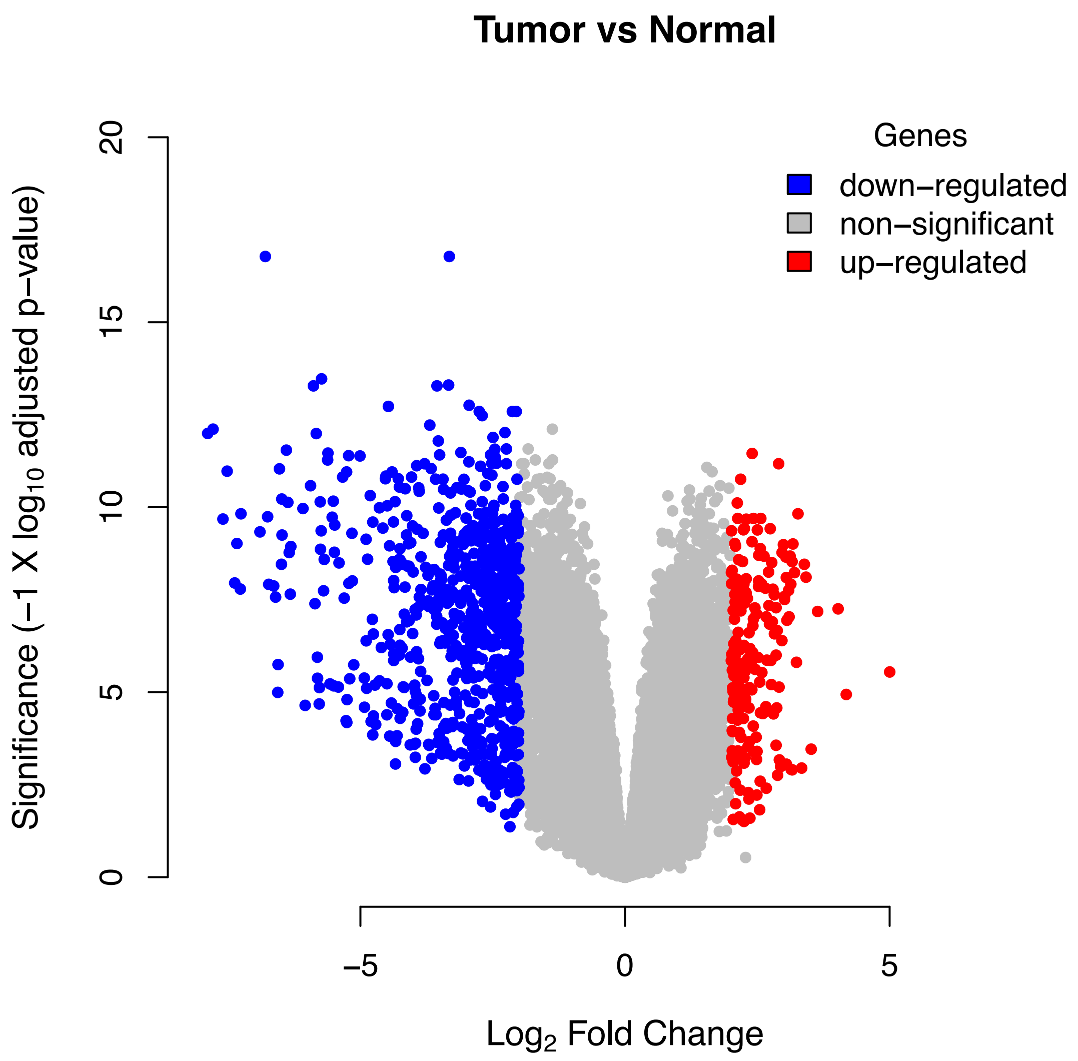

Supplementary Figure 8. Volcano plot of differentially expressed genes between osteosarcoma and normal bone samples.

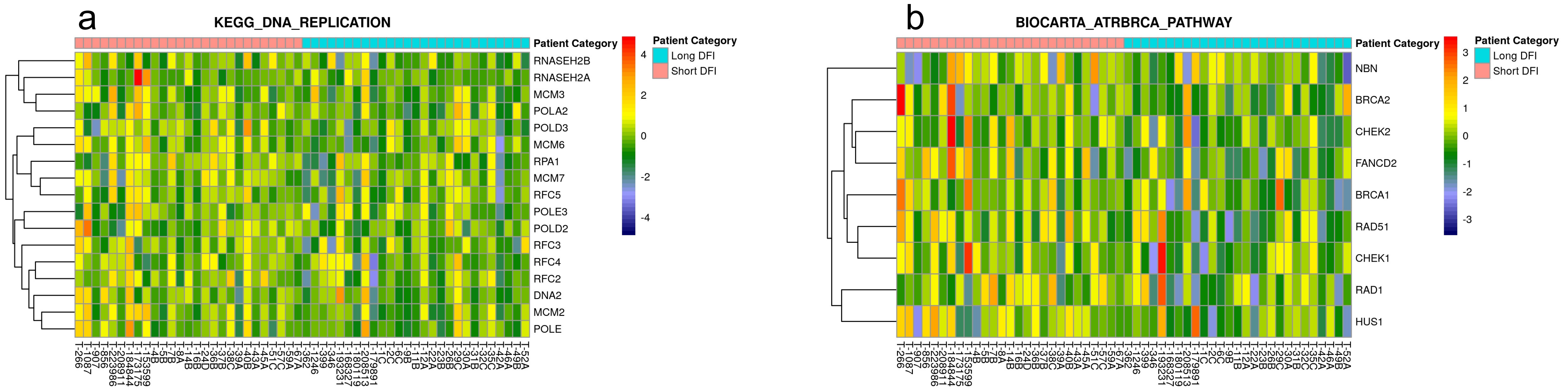

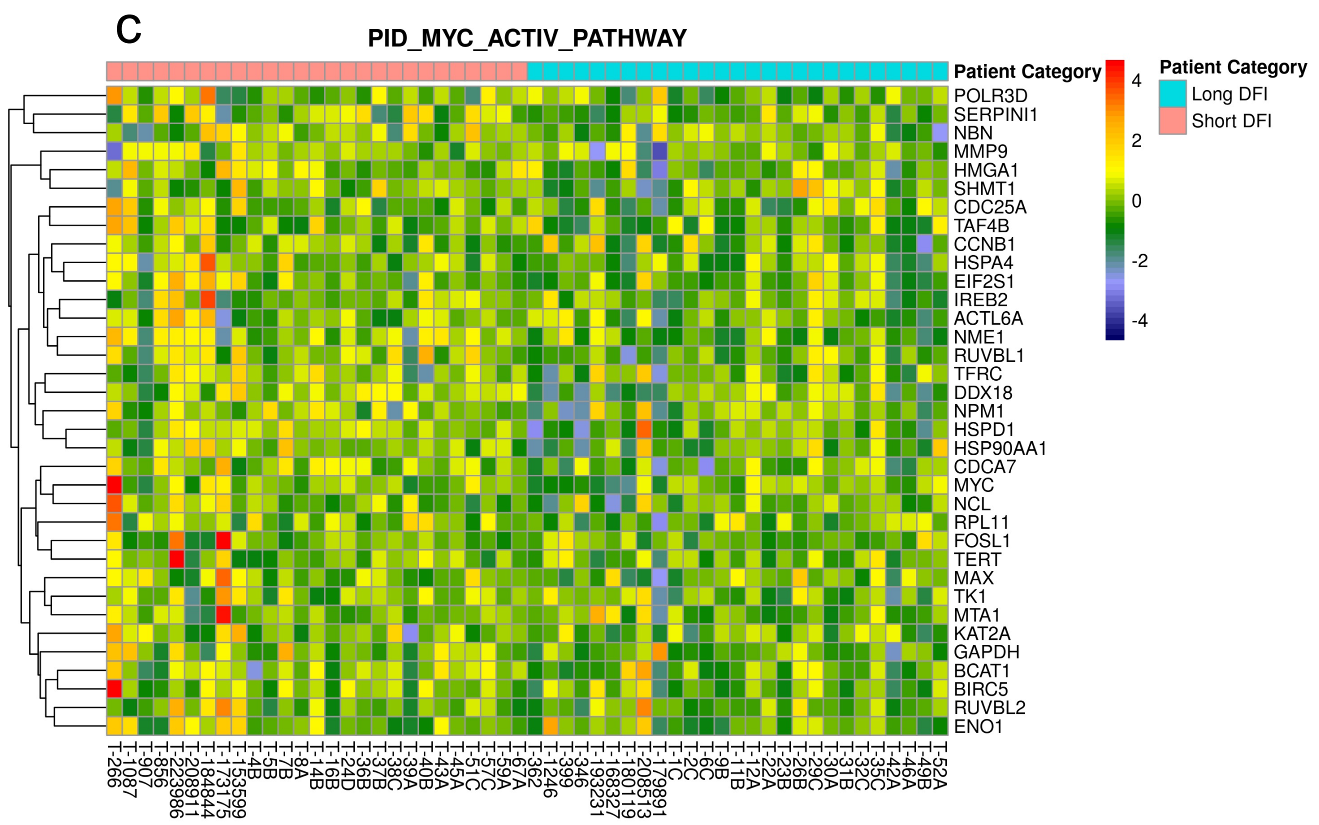

Supplementary Figure 9. Heatmap of gene expression enriched in short DFI patients. **a.** These genes are part of **b.** DNA replication; B. ATR BRCA pathways and **c.** Genes included in the MYC activation pathway. These gene sets were enriched in short DFI patients, that is, they had relatively higher expression in short DFI patients compared to long DFI patients. The heatmap scalebar represents row z-score.

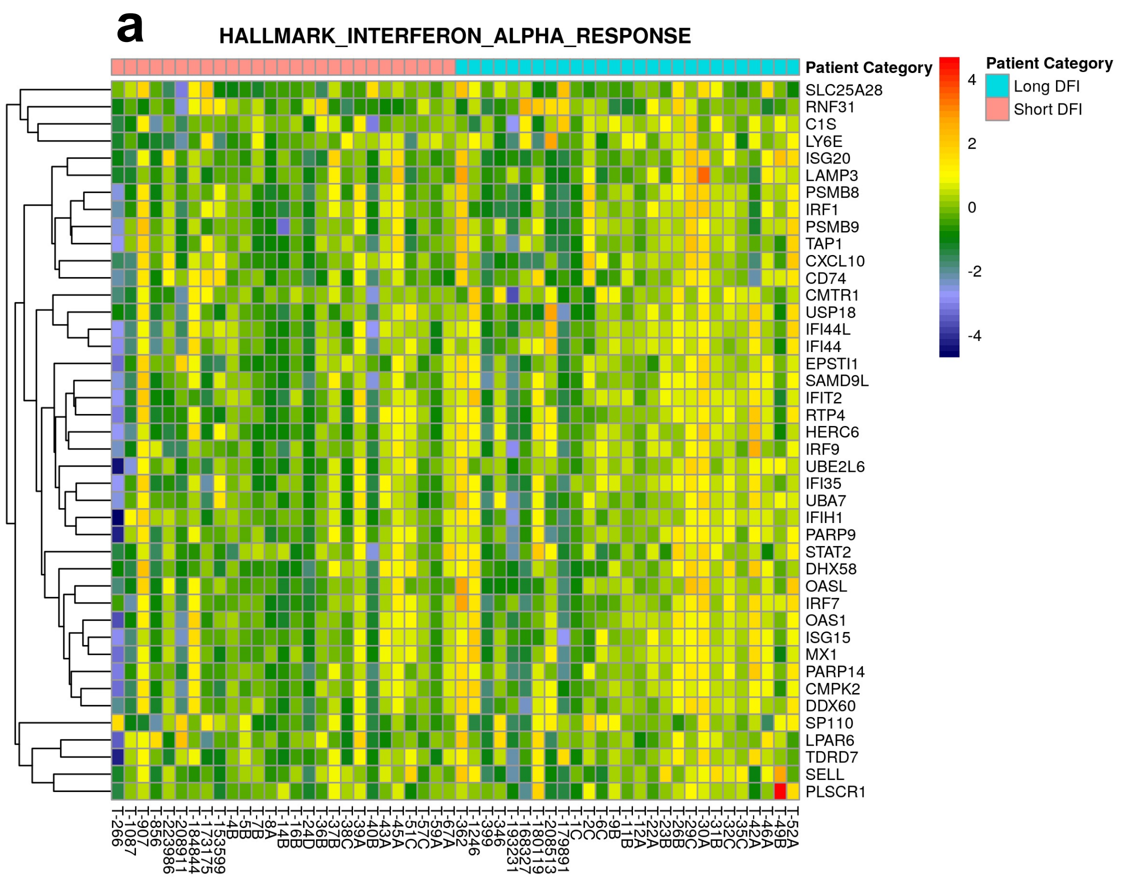

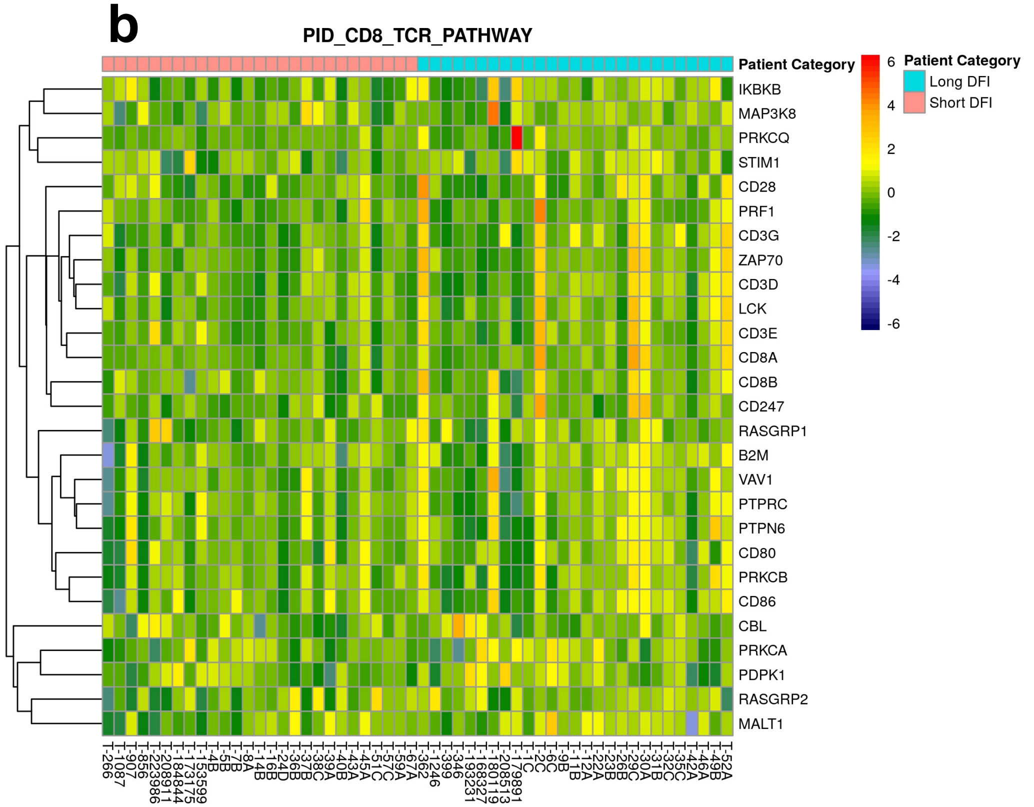

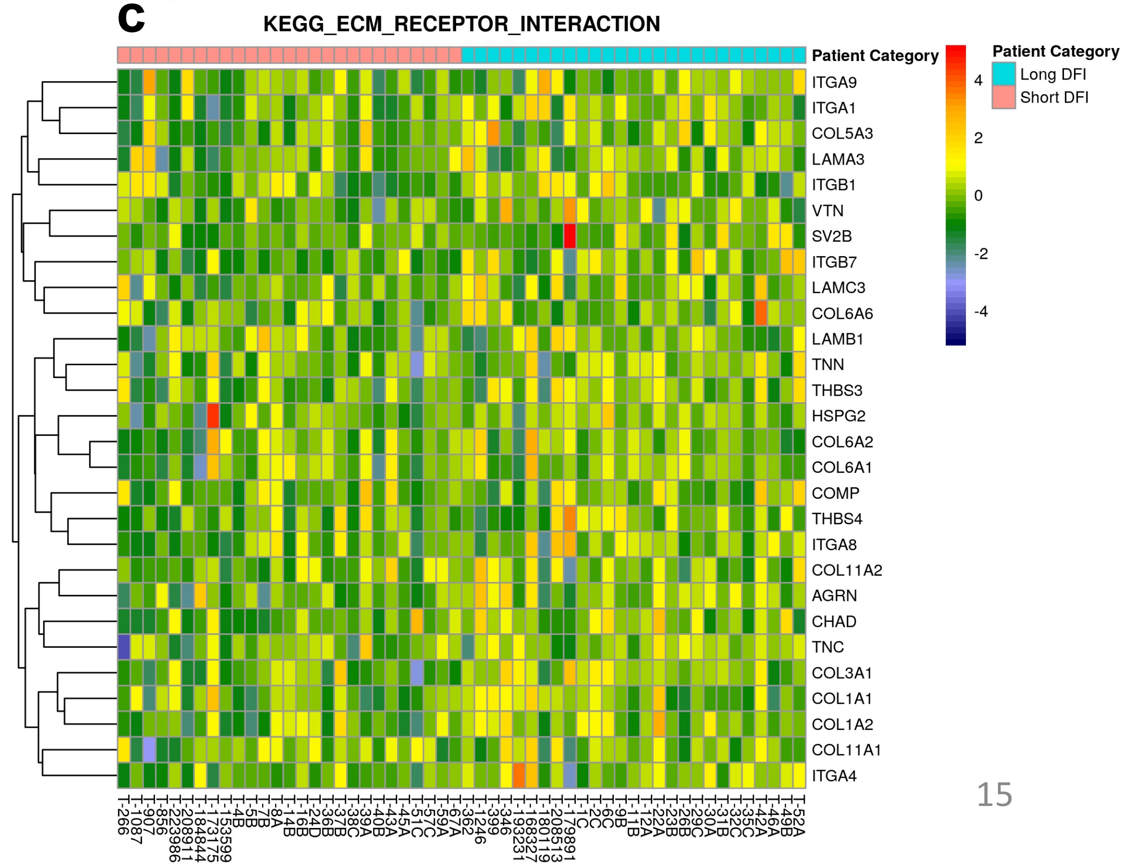

Supplementary Figure 10. Heatmap of gene expression enriched in long DFI patients. Figures a and b represent immune response gene sets. The genes in figure c are part of the ECM receptor interaction gene set. All these gene sets have relatively higher expression in long DFI patients when compared to short DFI patients. The heatmap scalebar represents row z-score.

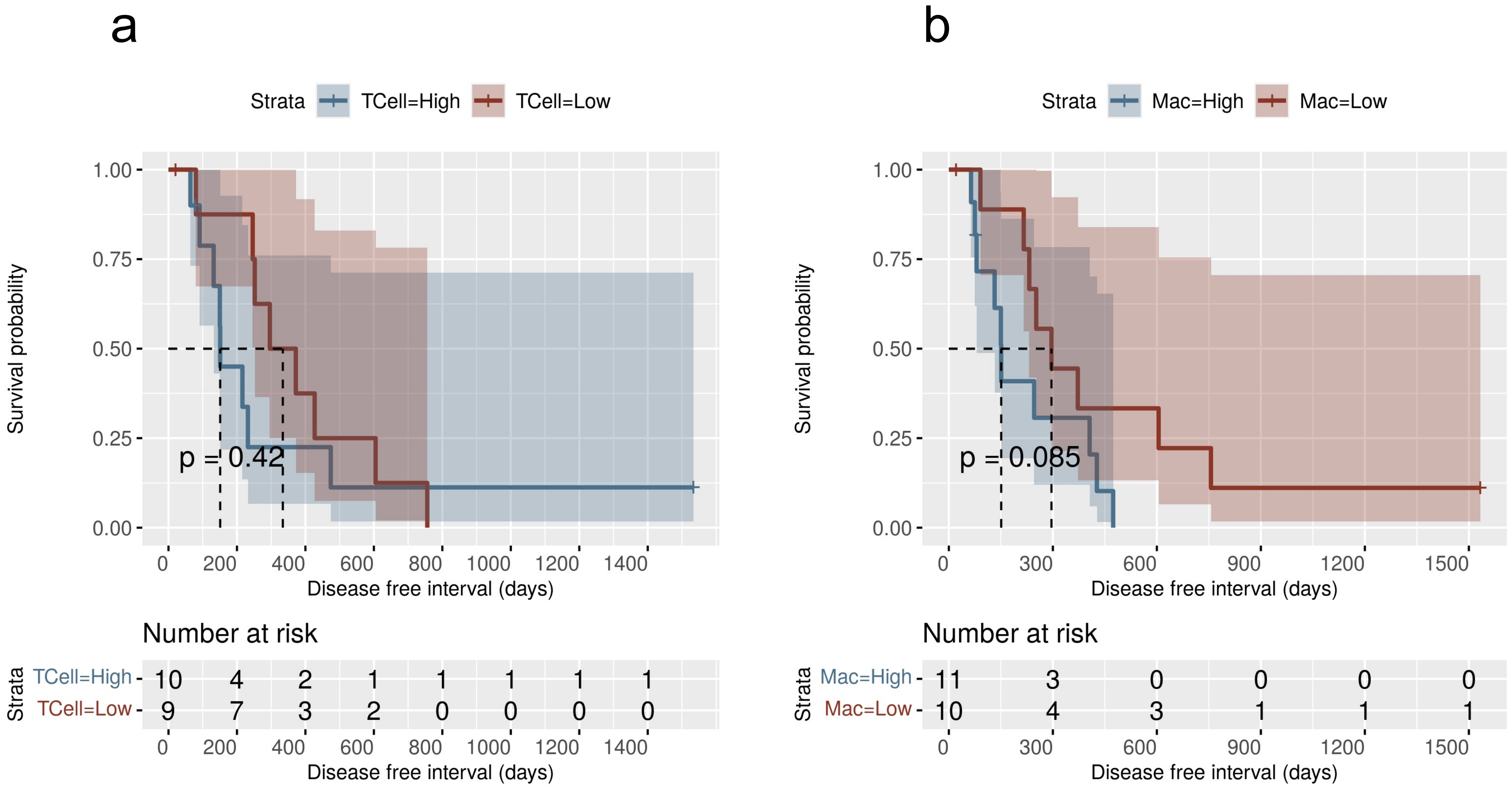

Supplementary Figure 11. Kaplan Meier plots. Survival plots in dogs with osteosarcoma were stratified by percent tumor area positive for CD3+ T-cells (a) and MAC387+ Macrophages (b). The red and blue lines represent high and low levels of T-cells or Macrophages (median dichotomized), respectively. The time event analyzed here is disease-free interval (DFI). Source data for these plots are included in the Supplementary Data 11.

Supplementary Figure 12. Heatmap of immune gene expression associated with 15 types of immune cell types or responses. The T-cell and macrophage infiltration was quantified via immunohistochemistry. The black colored samples on immune cell infiltration annotation bars represent the samples that were not available for immunohistochemistry analysis.

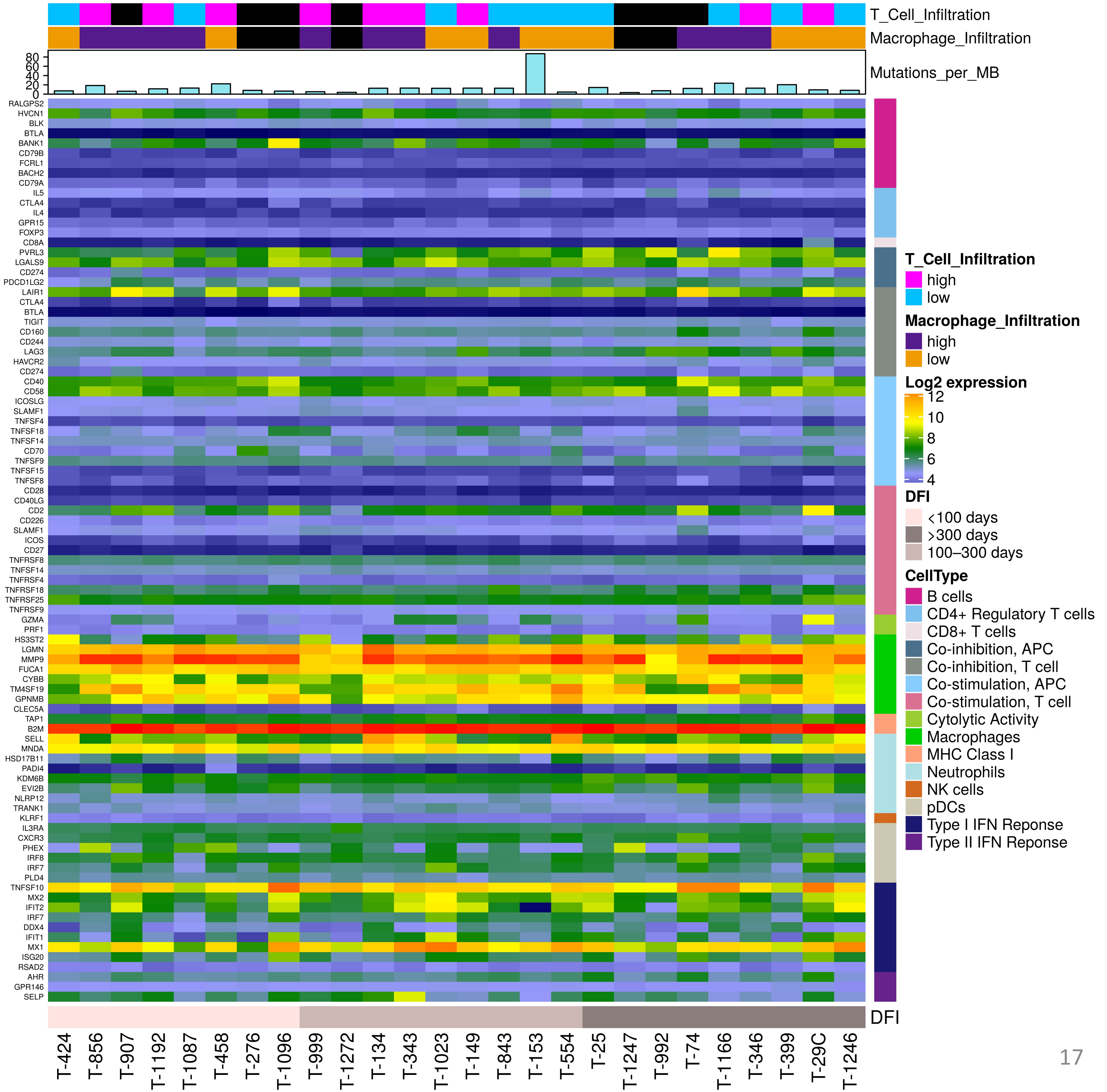

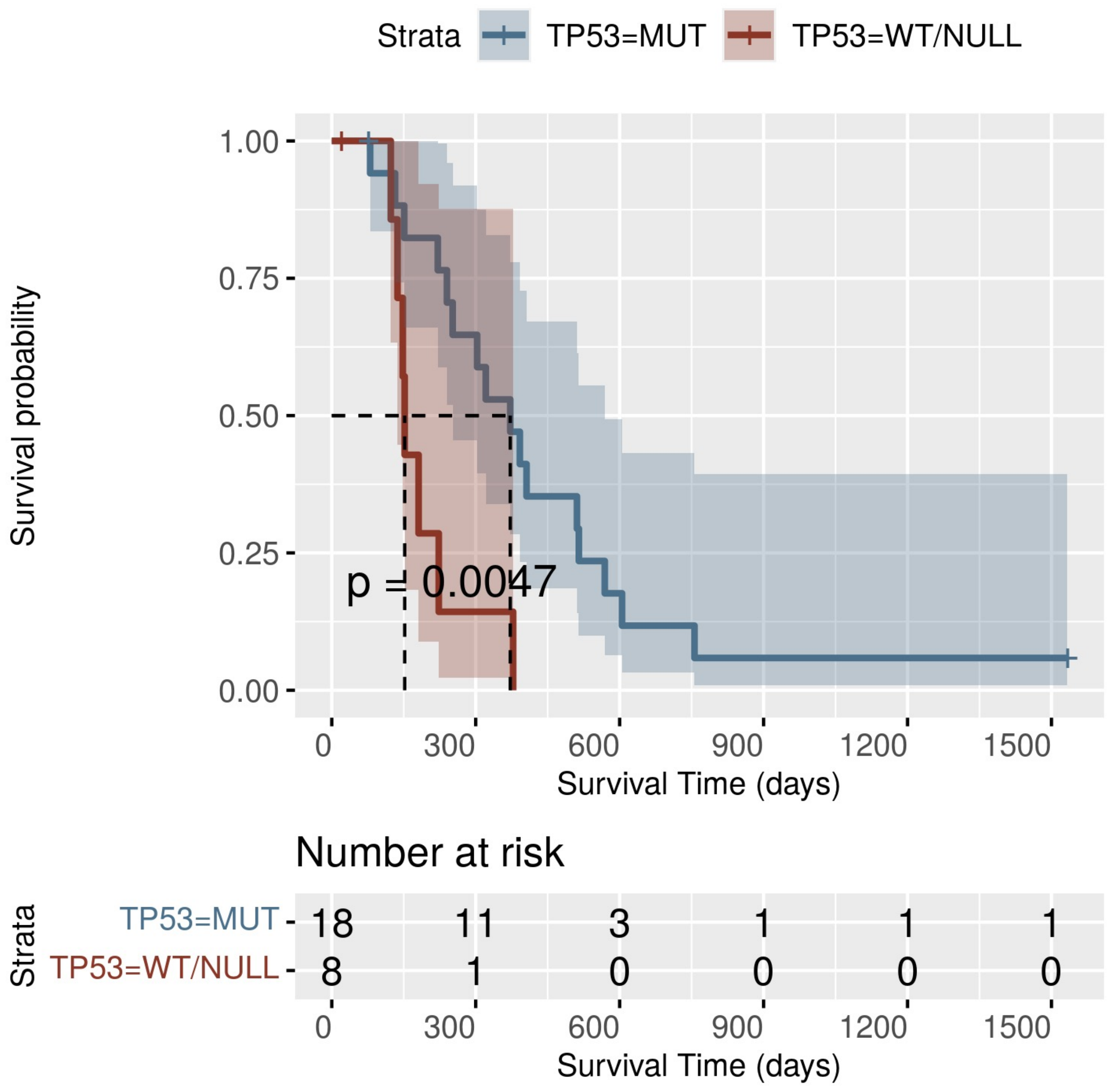

Supplementary Figure 13. Kaplan Meier plot using survival time. Stratification of patients were based on Wildtype/NULL and mutant *TP53* carrying missense variants. The time event analyzed here is survival time. Source data for this plot is included in the Supplementary Data 11.

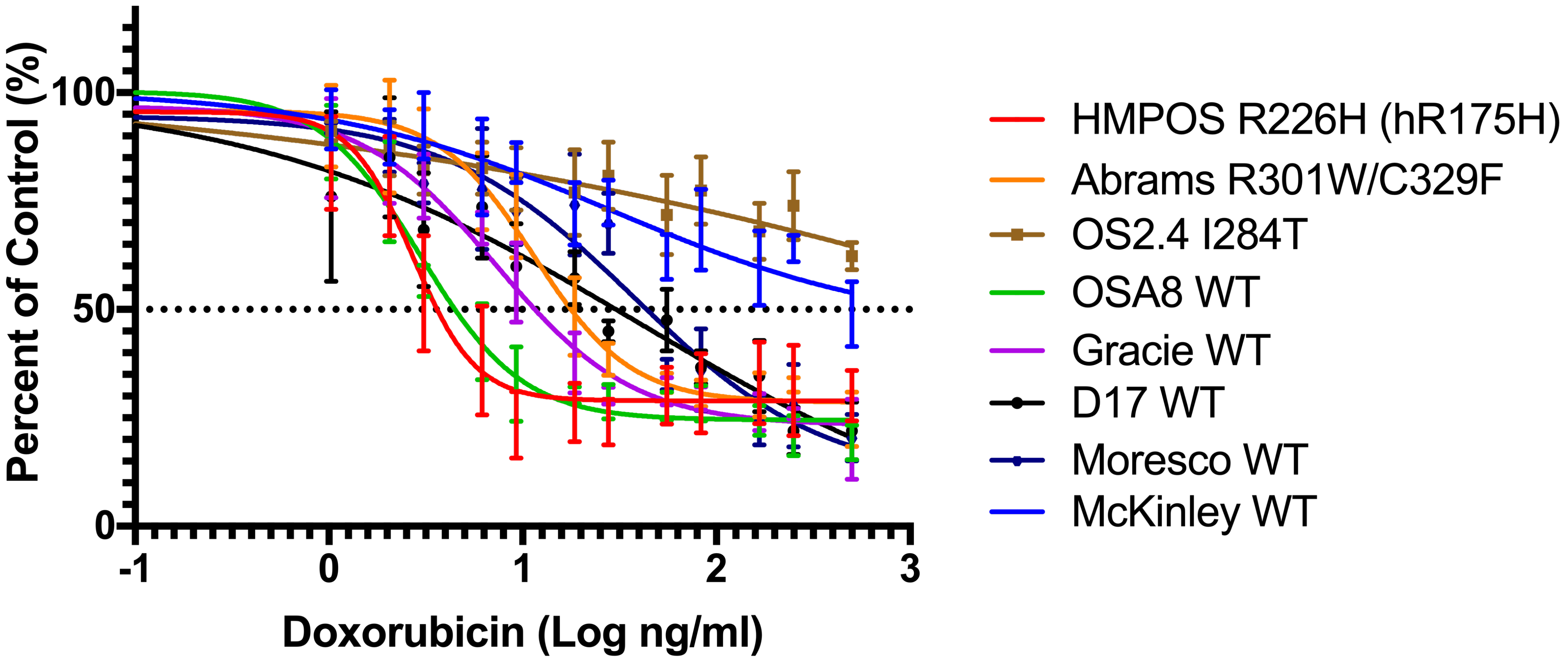

Supplementary Figure 14. Drug sensitivity assay of canine OSA cell lines to doxorubicin. Cells were plated on 96 well plates and treated with serial dilutions of doxorubicin 24 hours later. After treatment for 72 hours, cell viability was assessed with a resazurin-based fluorescent assay, normalized to time zero readings, and expressed as percent of control. Cell lines are annotated with identified *TP53* missense mutations. The HMPOS cell line bears an R226H variant homologous to the human R175H hotspot mutation and exhibits the greatest sensitivity to doxorubicin. The error bars are mean ±SD (n=3 to 4 independent experiments). Source data for this plot is included in the Supplementary Data 11.

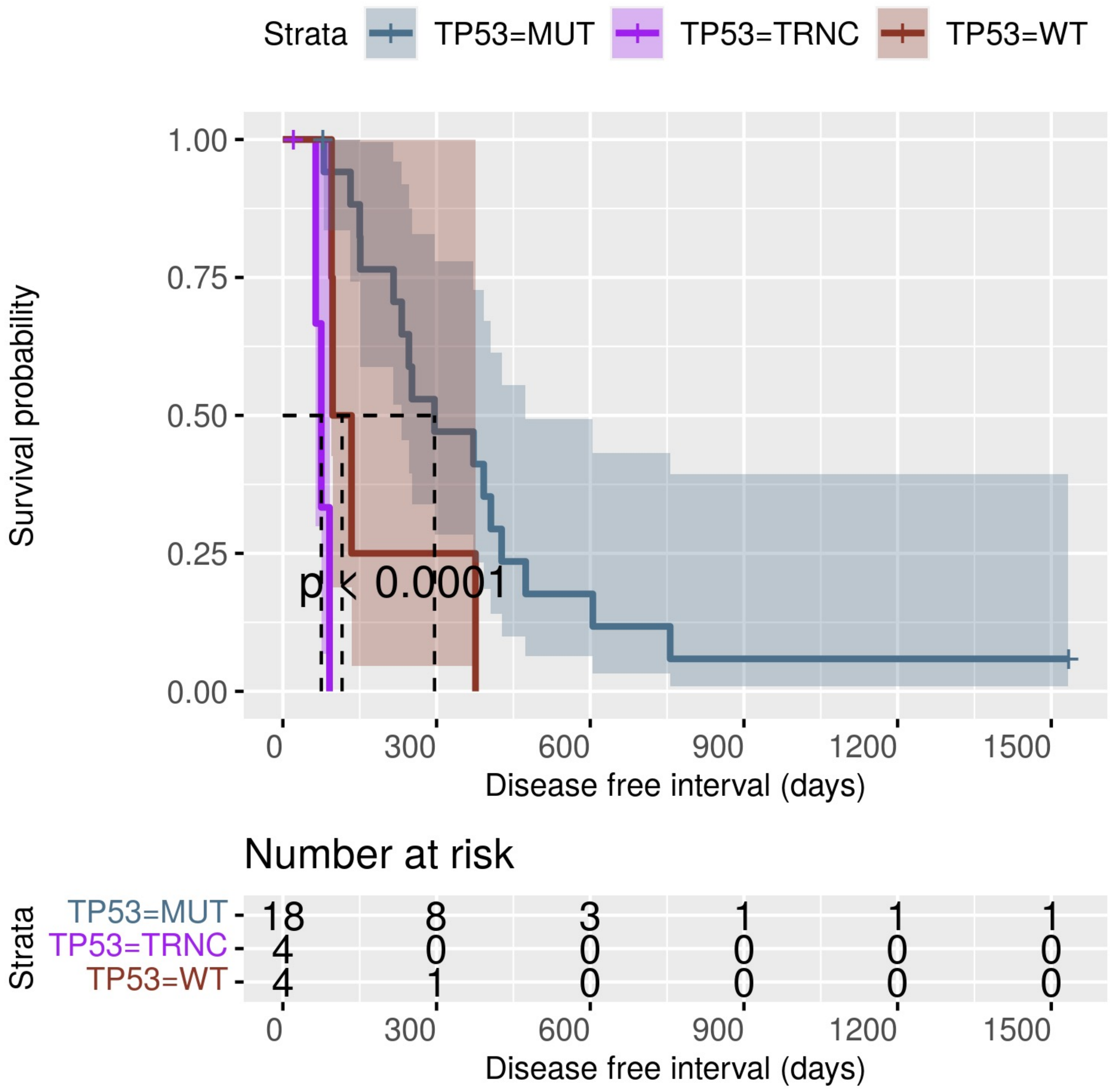

Supplementary Figure 15. Kaplan Meier plot using three types of TP53 mutation status. Stratification of patients were based on presence of either wildtype (WT), or truncation (TRNC) or missense (MUT) mutations within *TP53* gene. The time event analyzed here was disease free interval. Source data for this plot is included in the Supplementary Data 11.

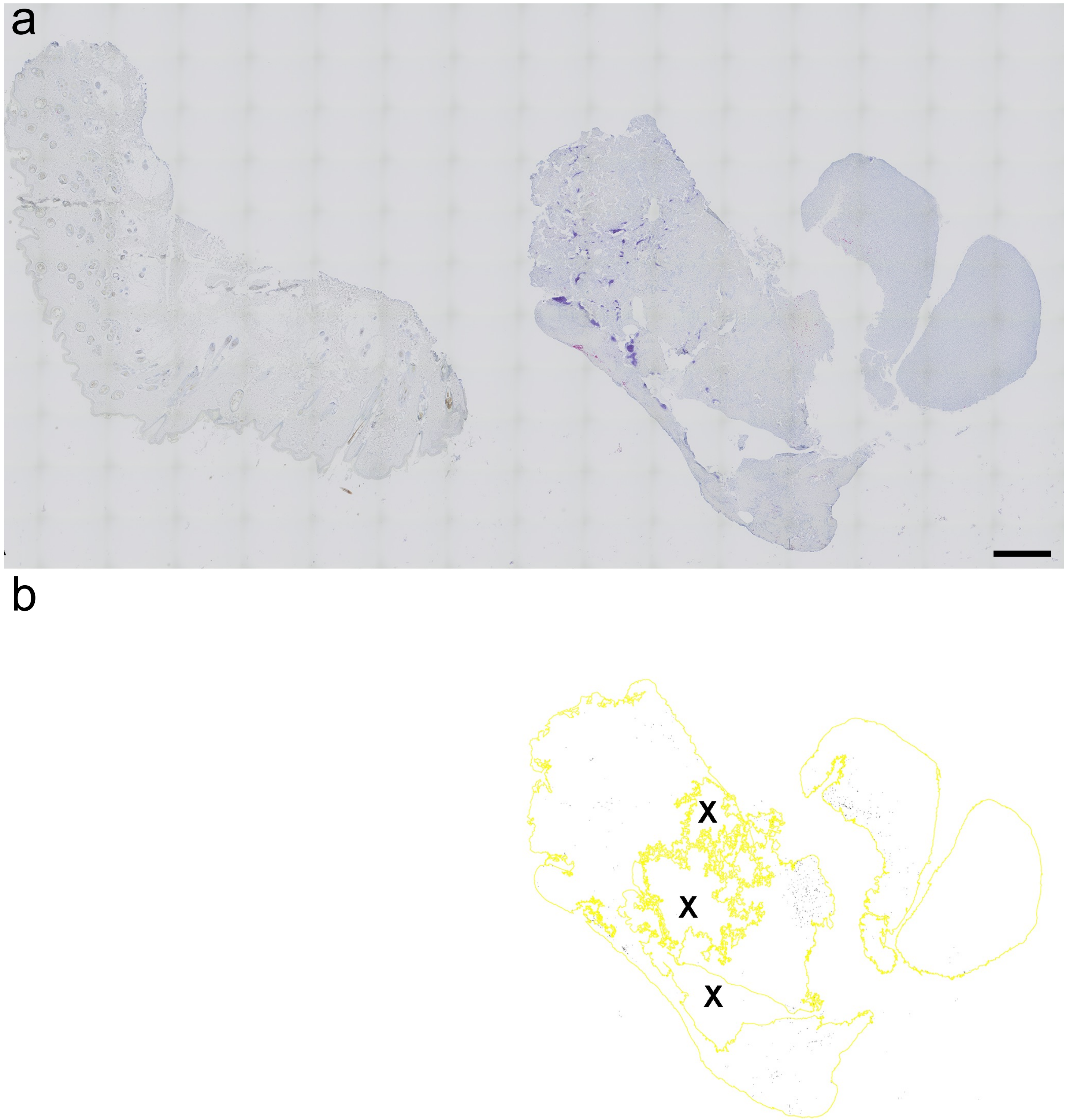

Supplementary Figure 16. Quantification of tumor area of interest. a. Parent 10x magnification whole-slide image of case 424 immunolabeled for CD3. Fast Red chromogen. Hematoxylin counterstain. Scale bars = 1 mm. b. ImageJ image analysis mask for case 424. The tumor area analyzed is outlined in yellow and CD3 positive cells are pseudo-colored black. Note the large section of normal haired skin removed from the analysis. X's denote regions of tumor containing abundant osteoid and which were excluded from the tumor area analysis.

Supplementary References

1 MacEwen EG, Kutzke J, Carew J, Pastor J, Schmidt JA, Tsan R, et al. c-Met tyrosine kinase receptor expression and function in human and canine osteosarcoma cells. Clin Exp Metastasis 2003;20:421-30

2 Riggs JL, McAllister RM, Lennette EH. Immunofluorescent studies of RD-114 virus replication in cell culture. J Gen Virol 1974;25:21-9

3 O'Donoghue LE, Rivest JP, Duval DL. Polymerase chain reaction-based species verification and microsatellite analysis for canine cell line validation. J Vet Diagn Invest 2011;23:780-5

4 F. Barroga E, Kadosawa T, Okumura M, Fujinaga T. Establishment and Characterization of the Growth and Pulmonary Metastasis of a Highly Lung Metastasizing Cell Line from Canine Osteosarcoma in Nude Mice. Journal of Veterinary Medical Science 1999;61:361-7

5 Mealey KL, Barhoumi R, Rogers K, Kochevar DT. Doxorubicin induced expression of P-glycoprotein in a canine osteosarcoma cell line. Cancer Lett 1998;126:187-92

6 Thomas R, Wang HJ, Tsai PC, et al. Influence of genetic background on tumor karyotypes: evidence for breed-associated cytogenetic aberrations in canine appendicular osteosarcoma. Chromosome Res. 2009;17(3):365-377.

Supplementary Note 1. Javascript macro used for this analysis in ImageJ analysis. This script was used to measure the immunohistochemical sections to determine the percentage of tumor stained with antibodies. See associated Supplementary Figure 16 for more details.

```
folder = getDirectory("Choose a Folder with Images");
setBatchMode(true);
listFiles(folder);
setBatchMode(false);
showMessage("Macro Complete");
 function listFiles(folder)
{
     list = getFileList(folder);
     for (i=0; i<list.length; i++)
     {
          //Do subfolders///
          if (endsWith(list[i], "/"))
          {
               listFiles(""+folder+list[i]);
          }
          else if (endsWith(list[i], ".tif")&&matches(list[i],
".*10x.*") || matches(list[i], ".*10X.*")) //
        {
             open(folder + list[i]);
             image = File.nameWithoutExtension;
             if (Overlay.size != 0 )
             {
                   run("To ROI Manager"); // For Saved Overlays
             }
               process(image,list[i],'');
               run("Close All");
               run("Collect Garbage");

        }
     }
}
function process(image,pic, quad)
{
     selectWindow(pic);
   //run("Add to Manager");//Saved Selection

   //run("Set Scale...", "distance=0.3125 known=1 pixel=1
unit=unit global");
   run("Set Scale...", "distance=0.7812 known=1 pixel=1 unit=µm
global");
   run("Colour Deconvolution", "vectors=[FastRed FastBlue DAB]
hide");
```

```
   selectWindow(pic+"-(Colour_3)");
     setThreshold(0, 135);
     run("Analyze Particles...", "size=10-Infinity show=Masks");
     rename("Melanin Mask");
     run("Dilate");
     selectWindow(pic+"-(Colour_3)");
     close();
     selectWindow(pic+"-(Colour_2)");
     close();
     selectWindow(pic+"-(Colour_1)");
     //getRawStatistics(nPixels, mean, min, max, std);
     setThreshold(0, 150);
     run("Analyze Particles...", "size=10-Infinity show=Masks");
     rename("CD3 Mask");
     selectWindow(pic+"-(Colour_1)");
     close();
     imageCalculator("Subtract create", "CD3 Mask","Melanin
Mask");
     selectWindow("Melanin Mask");
     close();
     selectWindow("CD3 Mask");
     close();
     selectWindow("Result of CD3 Mask");
     rename(image+','+quad);


     //fill selection here
     if(roiManager("count") != 0)
     {
          setForegroundColor(255, 255, 255);//white
          roiManager("fill");
          //roiManager("Set Line Width", 2);
          //roiManager("Draw");
     }

     run("Analyze Particles...", "pixel size=10-Infinity
show=Masks summarize");
     rename("cd3");
     selectWindow(image+','+quad);
     close();
     selectWindow(pic);
     run("Select None");
     run("Duplicate...", "title=BW");
     selectWindow("BW");
     run("8-bit");
     run("Find Edges");
     //setAutoThreshold("MinError dark");
```

```
        if(roiManager("count") != 0)
        {
             setForegroundColor(0, 0, 0);//black
             roiManager("fill");
        }
        setThreshold(15,255);
        run("Analyze Particles...", "pixel size=100-Infinity
show=Masks");
        selectWindow("BW");
        close();
        selectWindow("Mask of BW");
        run("Fill Holes");
        if(roiManager("count") != 0)
                  {
                       setForegroundColor(255, 255, 255);//white
                       roiManager("fill");
                       roiManager("Set Line Width", 2);
                       roiManager("Draw");
                  }

        if(roiManager("count") != 0)
        {
             roiManager("Delete");
        }

        run("Analyze Particles...", "pixel size=50000-Infinity
show=Masks add");
        rename(image +" Tissue Mask,"+quad);
        setThreshold(15,255);
        run("Measure");
        selectWindow("Mask of BW");
        close();
        selectWindow(image +" Tissue Mask,"+quad);
        close();
        selectWindow("cd3");
        run("RGB Color");
        setForegroundColor(255, 0, 0);
        roiManager("Set Line Width", 5);
        roiManager("Draw");
        if(roiManager("count") != 0)
        {

             roiManager("Delete");
        }
        saveAs("Jpeg", folder+ image+" QC "+quad+".jpg");
        close();
        }
```
