# Supplementary material for: Immune pathways and TP53 missense mutations are associated with longer survival in canine osteosarcoma
Source: Commun Biol. 2021 Oct 11;4:1178. doi: 10.1038/s42003-021-02683-0 (PMC8505454; doi:10.1038/s42003-021-02683-0)
Supplement: Supplementary file 3 — Description of Additional Supplementary Files [file 42003_2021_2683_MOESM3_ESM.pdf]

Description of Additional Supplementary Files

File name: Supplementary Data 1-10
Description:

Supplementary Data 1: General description and sequencing statistics on normal and osteosarcoma samples used for whole exome sequencing study.

Supplementary Data 2: Clinical data on osteosarcoma canine patients.

Supplementary Data 3: List of protein coding variants across 26 osteosarcoma samples.

Supplementary Data 4: Functional annotation of the genes with somatic short variants.

Supplementary Data 5: The somatic short variants within TP53 coding sequence in canine osteosarcoma tumors and their corresponding human homologous variants that are oncogenic.

Supplementary Data 6: GISTIC2.0 annotated significantly amplified and deleted genes.

Supplementary Data 7: Functional annotation of the genes with copy number variations.

Supplementary Data 8: Significantly correlated genes between CNV amplitude and its expression levels.

Supplementary Data 9: Significantly enriched gene sets in tumors when compared to normal bone gene expression.

Supplementary Data 10: Significantly enriched gene sets in dogs with long disease-free interval when compared short disease-free interval.

File name: Supplementary Data 11.
Description: Excel file containing source data for: Fig. 1a, Fig. 1b, Fig. 1c, Fig. 1d, Fig. 4e-f, Fig. 5b, Fig. 5c, Fig. 6a-b, Supplementary Figure 1, Supplementary Figure 2 and 3, Supplementary Figure 4a, Supplementary Figure 4b, Supplementary Figure 7, Supplementary Figure 11, Supplementary Figure 13, Supplementary Figure 14, Supplementary Figure 15.
