# Supplementary material for: Immune pathways and TP53 missense mutations are associated with longer survival in canine osteosarcoma
Source: Commun Biol. 2021 Oct 11;4:1178. doi: 10.1038/s42003-021-02683-0 (PMC8505454; doi:10.1038/s42003-021-02683-0)
Supplement: Supplementary file 6 — Reporting Summary [file 42003_2021_2683_MOESM6_ESM.pdf]

nature research

Corresponding author(s): Dawn L. Duval
Sunetra Das

Last updated by author(s): Aug 29, 2021

Reporting Summary

Nature Research wishes to improve the reproducibility of the work that we publish. This form provides structure for consistency and transparency in reporting. For further information on Nature Research policies, see our Editorial Policies and the Editorial Policy Checklist.

Statistics

For all statistical analyses, confirm that the following items are present in the figure legend, table legend, main text, or Methods section.

n/a	Confirmed	
☐	☒	The exact sample size (*n*) for each experimental group/condition, given as a discrete number and unit of measurement
☐	☒	A statement on whether measurements were taken from distinct samples or whether the same sample was measured repeatedly
☐	☒	The statistical test(s) used AND whether they are one- or two-sided<br>*Only common tests should be described solely by name; describe more complex techniques in the Methods section.*
☐	☒	A description of all covariates tested
☐	☒	A description of any assumptions or corrections, such as tests of normality and adjustment for multiple comparisons
☐	☒	A full description of the statistical parameters including central tendency (e.g. means) or other basic estimates (e.g. regression coefficient) AND variation (e.g. standard deviation) or associated estimates of uncertainty (e.g. confidence intervals)
☐	☒	For null hypothesis testing, the test statistic (e.g. *F*, *t*, *r*) with confidence intervals, effect sizes, degrees of freedom and *P* value noted<br>*Give P values as exact values whenever suitable.*
☒	☐	For Bayesian analysis, information on the choice of priors and Markov chain Monte Carlo settings
☒	☐	For hierarchical and complex designs, identification of the appropriate level for tests and full reporting of outcomes
☐	☒	Estimates of effect sizes (e.g. Cohen's *d*, Pearson's *r*), indicating how they were calculated

Our web collection on statistics for biologists contains articles on many of the points above.

Software and code

Policy information about availability of computer code

Data collection	The whole exome DNA library was created and genomic exonic regions were captured using the Agilent SureSelect XT All Exon Canine V2 (part number: 931198, Santa Clara, CA) capture kit. This capture encompasses 43.45 Mb of canine exonic regions. The SureSelectXT Target Enrichment System for Illumina Paired-End Multiplexed Sequencing Library kit was used to create the genomic DNA library. The resultant library was sequenced on an Illumina HiSEQ4000 sequencer generating 151 bp paired end reads. RNA samples with a RIN value >8 were analyzed on GeneChip Canine 2.0 Genome Arrays (Affymetrix).
Data analysis	The tools, databases, and their versions, in addition to the bioinformatic pipeline used in this study can be accessed here: https://github.com/sdas2019/Canine-Osteosarcoma-Whole-Exome-Sequencing-Pipeline.

For manuscripts utilizing custom algorithms or software that are central to the research but not yet described in published literature, software must be made available to editors and reviewers. We strongly encourage code deposition in a community repository (e.g. GitHub). See the Nature Research guidelines for submitting code & software for further information.

Data

Policy information about availability of data

All manuscripts must include a data availability statement. This statement should provide the following information, where applicable:

- Accession codes, unique identifiers, or web links for publicly available datasets
- A list of figures that have associated raw data
- A description of any restrictions on data availability

The Illumina raw fastq files were submitted to NCBI Short Read Archive (SRA) database. The canine osteosarcoma and normal samples have been submitted to Bioproject PRJNA613479 and PRJNA503860, respectively. The microarray data can be downloaded from Gene Expression Omnibus (GEO) database using accession number GSE76127 and GSE180303.

Field-specific reporting

Please select the one below that is the best fit for your research. If you are not sure, read the appropriate sections before making your selection.

☒ Life sciences ☐ Behavioural & social sciences ☐ Ecological, evolutionary & environmental sciences

For a reference copy of the document with all sections, see nature.com/documents/nr-reporting-summary-flat.pdf

Life sciences study design

All studies must disclose on these points even when the disclosure is negative.

Sample size	The sample numbers included in this study were based on available funding to sequence approximately 25 canine osteosarcomas and theavailability of gene expression microarray data, matched normal genomic DNA, and outcome data.
Data exclusions	The whole exome sequencing data for the T1166 matched metastatic tumor sample, M1166, was excluded from primary tumor analysis.Samples for which FFPE blocks were unavailable were excluded from immunohistochemical analysis.
Replication	Sanger sequencing of PCR amplified genomic DNA to confirm the presence of the TP53 variants in these tumors was conducted withsuccessful validation of the identified variants.
Randomization	This study is primarily descriptive so no randomization was conducted, samples were selected based primarily upon availability as describedpreviously without selection based on any of the criteria examined in this study.
Blinding	Pathological review of the immunohistochemical analysis was conducted by an investigator blinded to the other elements evaluated in thisstudy including mutational burden, disease free interval, variant identification, or gene expression data.

Reporting for specific materials, systems and methods

We require information from authors about some types of materials, experimental systems and methods used in many studies. Here, indicate whether each material, system or method listed is relevant to your study. If you are not sure if a list item applies to your research, read the appropriate section before selecting a response.

Materials & experimental systems

n/a	Involved in the study	
☐	☒	Antibodies
☐	☒	Eukaryotic cell lines
☒	☐	Palaeontology and archaeology
☐	☒	Animals and other organisms
☒	☐	Human research participants
☐	☒	Clinical data
☒	☐	Dual use research of concern

Methods

n/a	Involved in the study	
☒	☐	ChIP-seq
☒	☐	Flow cytometry
☒	☐	MRI-based neuroimaging

Antibodies

Antibodies used	Immunohistochemistry was performed via routine, automated methods on the Leica Bond Max autostainer (Leica Biosystems Inc.), with the following panel of previously published canine cross-reactive antibodies: mouse monoclonal anti-human CD3 (pan T lymphocyte marker; Leica, clone LN10, ready-to-use format), and monoclonal mouse anti-human Myeloid/Histiocyte antigen (MAC387; monocytes/macrophages; Dako, clone MAC387, 1:300 dilution/0.76 mg/mL).
Validation	Dako clone A0452 (Agilent Clone F7.2.38) has been utilized extensively in a wide variety of species with extensive information available regarding its utilization in other species including canine https://www.labome.com/product/Dako/A0452.html. In the current manuscript, we confirmed that CD3 staining with this antibody correlated with CD3 transcript expression in screened canine samples. Monoclonal mouse anti-human Myeloid/Histiocyte antigen (MAC387; monocytes/macrophages; Dako, clone MAC387) has also been used extensively and validated for use in canine samples https://www.labome.com/product/Dako/M0747.html.

Eukaryotic cell lines

Policy information about cell lines

Cell line source(s)	Cell Line Abrams: CSU/UWM;<br>Cell Line D-17: ATCC;<br>Cell Line Gracie: UWM;<br>Cell Line HMPOS: Tokyo;<br>Cell Line McKinley: CSU;

Cell Line Moresco: UWM;
Cell Line OS2.4: WSU;
Cell Line OSA8: UM

Authentication	Multiplex PCR was conducted and STR fragment sizes were determined and compared against a database of 59 canine cell lines. These cells were unique from other cell lines using a minimum cut off of 70% homology with the exception of the cell lines which were identical. A multiplex PCR for six common cell line species (human, chinese hamster, cat, rat, mouse, and dog) was conducted on isolated genomic DNA and these cell lines were determined to be of canine origin and free from contaminating species.
Mycoplasma contamination	All cell lines used in this study tested negative for mycoplasma contamination.
Commonly misidentified lines (See ICLAC register)	NA

Animals and other organisms

Policy information about studies involving animals; ARRIVE guidelines recommended for reporting animal research

Laboratory animals	This study did not involve laboratory animals.
Wild animals	This study did not involve laboratory animals.
Field-collected samples	This study did not involve laboratory animals.
Ethics oversight	Tumor and matched normal tissue samples were collected through the Colorado State University Flint Animal Cancer tumor bio-repository program with IACUC approval and informed consent.

Note that full information on the approval of the study protocol must also be provided in the manuscript.

Clinical data

Policy information about clinical studies

All manuscripts should comply with the ICMJE guidelines for publication of clinical research and a completed CONSORT checklist must be included with all submissions.

Clinical trial registration	While clinical outcome data is included in this study, the patient samples included here were obtained during clinical treatment for canine osteosarcoma and were not recruited specifically for this study.
Study protocol	Samples for this study ere collected following surgery to amputate the effected limb or during clinical treatment and this study had no impact on patient treatment.
Data collection	Samples were collected by the Colorado State University tumor bio-repository program program between 2004 and 2017.
Outcomes	Patients were determined to be disease free at amputation and Disease Free Interval was determined based on the [resence of lung metastasis observed in thoracic scans conducted periodically during and following treatment.
